# Letter to editor: careful literature search and exact data extraction are milestones of a meta-analysis: the case of dairy consumption and hip fracture

**DOI:** 10.1186/s12889-018-6175-1

**Published:** 2018-11-13

**Authors:** Hanieh Malmir, Ahmad Esmaillzadeh

**Affiliations:** 1 Students’ Scientific Research Center, Tehran University of Medical Sciences, Tehran, Iran; 2 Obesity and Eating Habits Research Center, Endocrinology and Metabolism Molecular Cellular Sciences Institute, Tehran University of Medical Sciences, Tehran, Iran; 3 Department of Community Nutrition, School of Nutritional Sciences and Dietetics, Tehran University of Medical Sciences, P.O. Box 14155-6117, Tehran, Iran; 4 Food Security Research Center, Department of Community Nutrition, Isfahan University of Medical Sciences, Isfahan, Iran

**Keywords:** Milk, Dairy, Hip fracture, Meta-analysis, Letter

## Abstract

In a recent issue of the BMC Public Health journal, Bian et al. described the results of an interesting meta-analysis on the association between dairy products consumption and risk of hip fracture. Although the results are important and valuable, some critical points should be noticed in the explanation of these findings. We prepared these critical points in a letter to the editor and hope to be an interest of you.

## Main text

In a recent issue of the journal, Bian et al. described the results of an interesting meta-analysis on the association between dairy products consumption and risk of hip fracture based on 18 observational studies [1]. They found that consumption of yogurt and cheese was associated with a lower risk of hip fracture. However, they failed to find any significant association between consumption of milk, cream and total dairy consumption and risk of hip fracture. Although, the results are important and valuable, some critical points should be noticed in the explanation of these findings.

First, as we all know, literature search is the most important step in any meta-analysis. Lack of inclusion of relevant studies might influence the whole findings of the meta-analysis. In the study of Bian et al., several publications have been missed and were not included [2–7]. For instance they did not include the study of Sahni et al., which assessed the relation between consumption of dairy and hip fracture in 3224 American adults (2). This is also the case for other studies. Second, although individual publications have reported covariate-adjusted RRs or ORs, the authors did not use these adjusted values and rather they applied crude RRs or ORs [8, 9]. Considering the contribution of other variables to hip fracture, it is very important to consider the independent association of dairy intake and risk of hip fracture. The third point is that most studies have reported RRs and ORs for hip fracture in the highest intake of dairy products versus the lowest intake. However, in three publications [10–12] Bian et al. included in the meta-analysis, the risk was reported per 1-cup or per a given grams of dairy consumption per day [10–12]. Bian et al. have combined these effect sizes, while we all know that combination of effect sizes, from studies that considered the exposure as categorical and continuous variables, can distort the overall findings. Finally, in two individual publications [11, 13], consumption of dairy products in childhood and adolescence period were linked with risk of hip fracture in adulthood [11, 13], while other included studies have examined dairy consumption in adulthood. Given the difference in dairy consumption between adolescence and adulthood, pooling the findings of these studies might influence the overall findings.

In conclusion, there is a great value in summarizing the relation between consumption of dairy products and risk of hip fracture in a meta-analysis; however, in order to avoid any incorrect conclusions, careful search of literature and data extraction must be done.

## Abbreviations

OR: Odds ratio
RR: Relative risk
